# Robust SMC-PSS and AVR design: A grid connected solar concentrated OTEC system application

**DOI:** 10.1371/journal.pone.0295941

**Published:** 2023-12-22

**Authors:** Hussein Abubakr, Abderezak Lashab, Tarek Hassan Mohamed, Juan C. Vasquez, Josep M. Guerrero, Yasser Ahmed Dahab

**Affiliations:** 1 Centre for Research on Microgrids (CROM), AAU Energy, Aalborg University, Aalborg, Denmark; 2 Faculty of Energy Engineering, Department of Electrical Engineering, Aswan University, Aswan, Egypt; 3 College of Computing and Information Technology, Arab Academy for Science, Technology & Maritime Transport, Giza Governorate, Egypt; Vellore Institute of Technology, INDIA

## Abstract

This work analyzes the stability and performance of an offshore solar-concentrated ocean thermal energy conversion system (SC-OTEC) tied to an onshore AC grid. The OTEC is a system where electricity is generated using small temperature differences between the warm surface and deep cold ocean water. Existing control methods for SC-OTEC systems lack coordination, hindering dynamic stability and effective damping for the synchronous generator (SG). These methods struggle to quickly adapt to sudden disturbances and lack the capability to adequately reject or compensate for such disturbances due to complex control constraints and computational demands. To this regard, a control strategy combining sliding mode control (SMC) and a power system stabilizer (PSS) to improve the SC-OTEC dynamic stability and damping features for the SG. Moreover, an auxiliary secondary automatic voltage regulator is assembled with a non-linear exciter system to provide damping features. The proposed PID-PSS and secondary AVR controller gains are adaptively tuned using a modified whale optimization algorithm with the balloon effect modulation. The studied SC-OTEC is tested through MATLAB/Simulink under a severe 3*ϕ* short-circuit fault, solar radiation variations, and a change in surface seawater temperature as well as changes in local loads. The final findings approved that the proposed control strategy preserves a strong performance and can mimic effectively the proposed SC-OTEC damping compared to the conventional system.

## 1. Introduction

During the Fourth Industrial Revolution, the global energy consumption has been on the rise, and it is expected to rise to 309.1 billion Btu by 2040. Therefore, renewable energy is predicted to experience significant growth, reaching 25.1 billion Btu, which represents a substantial increase of 44.2% [1]. Furthermore, there is a growing demand for renewable and new energy sources due to global environmental concerns, and the marine sector is being considered as a viable solution for renewable energy [2].

Ocean energy is a promising renewable energy source (RES) with a large amount of energy available due to the extensive coverage of seawater on Earth. It includes wave, tidal, wave, and ocean thermal energies. Ocean Thermal Energy Conversion (OTEC) is a process that harnesses ocean thermal energy to generate electricity [3]. It is another form of renewable energy that converts solar radiation into electrical energy [4,5]. By utilizing the natural thermal gradient of the ocean, OTEC facilitates the cycle of power production. However, OTEC has a relatively low thermal efficiency of around 3% to 4%, compared to other thermal power plants powered by nuclear or fossil fuels with efficiencies ranging from 30% to 43% [6]. Therefore, to ensure stable operation and improve OTEC efficiency, a comprehensive understanding of plant characteristics is essential to achieve a proper operating control [7]. Additionally, both deep and surface seawater temperatures significantly impact the OTEC plant power output. A temperature difference of about 20°C (36°F) between depths of 800–1000 meters enables the OTEC system to generate substantial power using a low-pressure turbine (LPT) [4,7]. Consequently, the oceans represent enormous RESs capable of providing billions of watts of electricity.

Further research is required to enhance the performance of the OTEC system, explore new cycles, establish demonstration plants, and design control systems. In [8], a novel control strategy for an OTEC plant with a double-stage Rankine cycle was proposed, considering variations in warm seawater temperature. This strategy effectively reduced the control characteristics of the two-stage OTEC system by using PI control in response to changes in warm seawater temperature. Another study [9] focused on stabilizing output and controlling the generation amount through governor control in different OTEC generation systems: open-cycle, closed-cycle, and hybrid-cycle. Additionally, a recent development study focused on the automatic control system for an OTEC demonstration plant using a sequence controller [10]. Its objectives were to analyze the manual start and stop process of a 20 kW-class pilot plant and ensure the safety of the system operation through the provision of a 1 MW-class design model that is currently under construction.

In [11], a detailed OTEC model was proposed to simulate a 20kWh latent heat volume with an average power of 10 kW. Offshore OTEC systems can be open cycle (using steam from seawater) or closed cycle (using fluids with low points of boiling). OTEC offers several advantages over other RESs, including the potential for electricity generation and power self-sufficiency in tropical regions [12]. Therefore, OTEC is considered a promising technology for the future. However, conventional SC-OTEC systems have limitations in terms of output control and vibration issues caused by the length of pipes. Additionally, there is significant energy waste since OTEC systems are located far from consumption sites. To tackle this challenge, the conversion of OTEC energy to hydrogen through electrolysis is being explored using RESs [13].

Several studies have been conducted on SC-OTEC systems, as follows. In [3,14], dynamic simulations were performed to analyze the system performance of SC-OTEC using PID automatic control. The aim was to improve the damping characteristics of the synchronous generator (SG) and implement power system stabilizer (PSS) control theory based on temperature changes through fluid circulation control. Furthermore, the governor and turbine models of SC-OTEC were linearized into first-order transfer functions, to develop a robust nonlinear fractional order proportional integral derivative (NLFOPID) controller for frequency regulation in restructured energy systems.

Previous scholars focused on the PSS as the main culprit in this problem. Aside from the PSS, an adaptive automatic voltage regulator (AVR) is introduced as a tool to deal with this challenge. To enhance the dynamic performance of the AVR, a standard PID regulator is commonly employed due to its simple design, robustness, and ease of implementation [15]. The suggested AVR system utilizes a linearized and simple model that considers the main time constants while avoiding non-linearities [16]. Therefore, this work focuses on the system voltage regulation using an auxiliary secondary AVR to control the SG voltage level in all operating conditions [17].

On the other hand, a sliding mode control (SMC) has earned a significant attention in control engineering due to its fast responsiveness. It is designed based on system parameters and perturbations, thereby enhancing the control strength of the sliding mode [18]. The sliding surface is a linear set of system states constructed with appropriate coefficients that are invariant with time. It is well known that implementing SMC requires specific switching surfaces and controller tuning [19]. By forcing the state trajectories to converge to the sliding mode surface within a given time, the transient system performance can be greatly improved. Thus, SMC has been widely applied in various applications [20–22].

Recently, soft techniques have been used for online tuning the system parameters to address system uncertainties [23]. In this paper, an enhanced version of whale optimizer (EWOA) [24] is employed. The algorithm has been tested in various applications, utilizing benchmark unimodal and multimodal functions. The evaluation involved statistical analysis using ANOVA, convergence tests, and comparison with other heuristic and metaheuristic optimizers, as detailed in [25]. The primary objective of employing this algorithm in the present study is to adaptively tune the secondary AVR and PID-PSS controller gains. It offers benefits such as simplicity and powerful. Unlike gradient-based algorithms that require updating gradients at each iteration, the enhanced WOA does not rely on gradient computations. However, a drawback of using classic algorithms in an adaptive manner is the poor performance during contingencies. This is due to their design based on the nominal plant transfer and consideration of no-load disturbance. To overcome this issue, the BE modulation is introduced to enhance the sensitivity of EWOA in handling system disturbances and uncertainties. The BE modulation concept has been successfully applied in various applications, as demonstrated in [26–28].

In this study, a dynamic SC-OTEC model connected to AC grid is demonstrated using the inclusion of SMC and BE modulation with PSS and secondary AVR control parts in adaptive manner. The developed model is suitable for transient simulations of a balanced three-phase power system. The main concept of associating the BE is to increase the EWOA optimizer sensitivity via system uncertainties. Using this type of control strategy for an offshore (OTEC) system tied to an onshore grid is considered for the first time.

The novelty of this work lies in the use of an auxiliary secondary AVR, which is applied for the first time, along with the non-linear exciter system in an adaptive manner. This arrangement provides suitable damping properties for the SG and deliver a complementary signal to promote faster oscillatory instability on the grid generation side. Furthermore, to handle variations in system parameters and improve the SC-OTEC dynamic response, SMC is combined with PSS and BE modulation to enable self-tuning of the secondary AVR and PID-PSS controller gains. Finally, this control strategy is validated through several contingencies such as 3*ϕ*-short circuit fault at the grid AC bus, variations in load and solar radiation, and changes in surface seawater temperature.

## 2. Working system dynamic model

**Fig 1:**
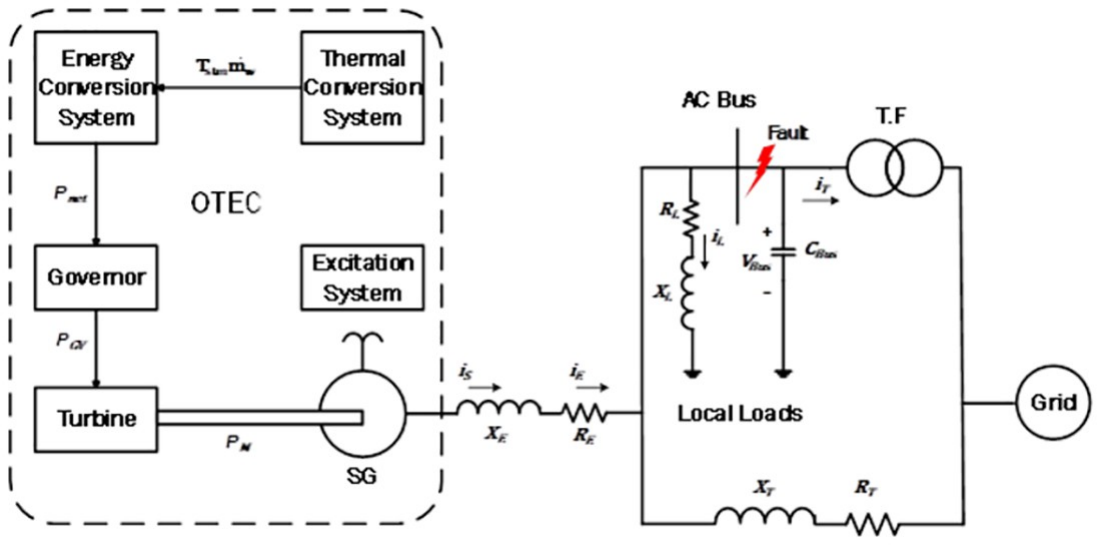
Grid-tied SC-OTEC system one-line diagram.

This work focuses on a closed SC-OTEC system, as shown in Fig 1, which is connected to the grid. The system is located offshore and connected to an onshore substation via undersea cables [29]. It consists of energy and thermal conversion systems, a governor and steam turbine, an excitation system-based SG, local loads, a capacitor bank linked with the AC bus, transmission lines, and an electrical grid. The SC-OTEC model and the corresponding differential equations are described in this study. The nominal parameters of the SC-OTEC and the single machine infinite bus are given in “Tables 1 and 2 in S1 Appendix”.

### 2.1. Thermal conversion system

This process involves a heat centralized tube, heat exchanger, and a parabolic trough. The parabolic mirror concentrates solar heat onto the tube, resulting in the heating of the flowing water inside the tube. The heat exchanger transfers the generated heat to the surface seawater, as described in [3]. The essential equation for heat absorption in the central tube can be expressed as:

$$q_{u}=A_{C}F_{R}\left[S\alpha \tau -U_{L}\left(T_{stm}-T_{a}\right)\right]$$ (1)

$$F_{R}=(\frac{m_{cs}^{\prime }c_{w}\;}{A_{C}U_{L}})\left[1-exp\left(\frac{A_{C}U_{L}F^{\prime \prime }}{m_{cs}c_{cs}}\right)\right]$$ (2)

and (*)′ = d(*)/dt

In order to find the equilibrium equation for the heat exchanger: the heat should be absorbed by the exchanger first, and then discharged it into surface seawater as:

$${(M}_{w}C_{w})p\left(T_{stm}^{\prime }\right)=q_{u}-{(U}_{s}A_{s}\left(T_{stm}-T_{a}\right))-m_{w}^{\prime }C_{p}\left(T_{stm}-T_{wx}\right)$$ (3)

### 2.2. Energy conversion system

The closed-cycle diagram of the OTEC system consists of two essential parts: the evaporator and the condenser. The former transmits heat to the working fluid, while the latter releases heat from it. The evaporator heat exchanger is responsible for converting liquid ammonia into a gaseous state.

The condenser heat exchanger cools down the ammonia vapor by pumping the deep cold seawater inside. This creates a pressure variance between the condenser and the evaporator, which drives the LPT blades. This mechanical energy is utilized by the SG to produce electricity, which can be transferred to the power grid through transmission lines. The net energy produced in this process dependent on *T*_*stm*_ and **$m_{w}^{\prime }$** as inputs and can be briefly put into an equation, as follows [3].


$$P_{net}=\frac{(m_{w}^{\prime }\rho )C_{p}3\mu [({T_{stm}-T_{c})}^{2}-0.3\left({{\Delta T}_{dseign})}^{2}\right]}{\left(16(1+\mu )(T_{stm}+273.15)\right)}$$ (4)

### 2.3. Energy conversion system

**Fig 2:**
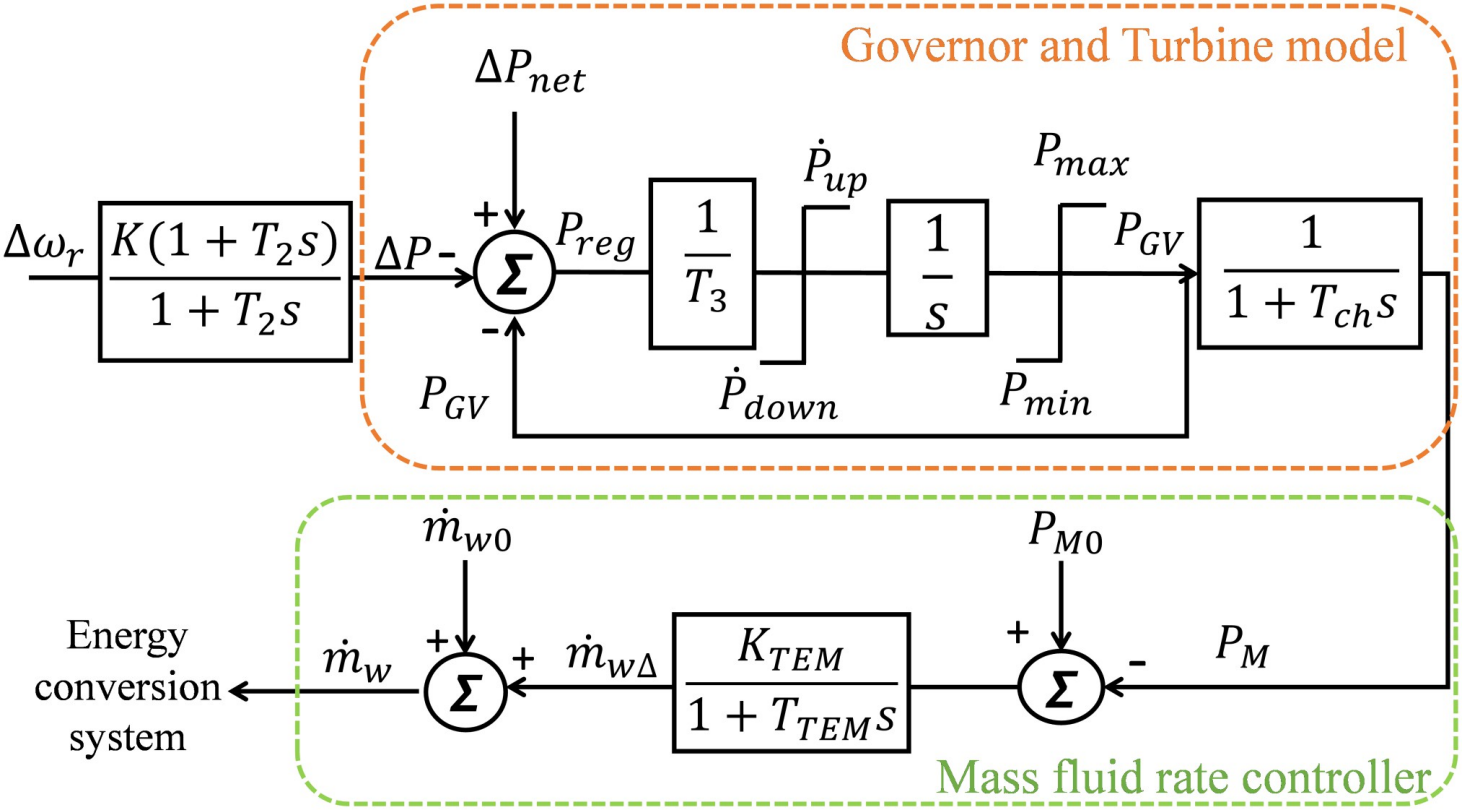
Turbine and governor block diagram with mass fluid rate controller.

The characteristics of OTEC turbine are determined by the type of working fluid used, which typically involves high mass flow and low-pressure ratios. Fig 2 illustrates a simplified model of the turbine and governor, excluding the steam feedback process [3]. Further details about governor and turbine modelling can be found in “S1 Appendix”.

### 2.4. Mass fluid rate controller

The mechanical power generated by the turbine is highly influenced by the heat exchanger’s average temperature and the warm seawater mass fluid rate. Therefore, controlling the flow rate is necessary to achieve the desired SG mechanical power, as shown in Fig 2. The flow rate controller in Fig 2 adjusts the rate of the flow based on the difference between the reference mechanical power (*P*_*Mo*_) and the measure level of power (*P*_*M*_) in pu.


$${{(T}_{TEM})\left({\Delta m}_{w}^{\prime }\right)}^{\prime }={(K}_{TEM}\Delta P_{M})-{\Delta m}_{w}^{\prime }$$ (5)

### 2.5. Synchronous generator and excitation system

SGs serves as the main provenance of electricity in power systems [30]. The excitation system control has a directly impact on the voltage stability. It generates DC voltage to establish current flow in the field windings of the generator. Therefore, there is a direct relationship between the terminal voltage and exciter of the generator, as discussed in section 4. More detailed information about the modelling of the SG and excitation system modelling can be found in the “S1 Appendix”.

## 3. EWOA and balloon effect modulation: Main concept

### 3.1. For EWOA optimizer

The classic WOA is inspired by humpback whale hunting behavior and introduced by [25]. The whales use a bubble net hunting strategy, creating bubbles in a spiral shape to encircle and capture prey. WOA assumes the prey’s current location is the best solution, and search agents update their positions accordingly. The algorithm has two paths: the shrinking encircling mechanism and the spiral moderating position. In the shrinking mechanism, the value of ’a’ decreases over iterations. In the spiral position, the whale and prey move in a helix-shaped path defined by a logarithmic spiral equation.

Whale movement is influenced by a probability factor. In the exploration phase, whales randomly search for prey based on the exploration vector |A|. If |A| is greater than or equal to 1, they search for the global optimum; if less than 1, they update their position. The algorithm is enhanced by adding an inertia weight (ω) to modify the searching process. The position vector of classic WOA is updated to a modernized version by multiplying it with the inertia weight. For more details about the prey strategy, movements of the whales, and updating their position, see [25]. A pseudo code using MATLAB is created to describe the whole idea of the EWOA, as follows.

Initialize the whale’s population (i = 1, 2,…, n)

Calculate the fitness of each search agent.

Identify the best search agent.

*while (t < maximum number of iterations)*

 for each search agent, Update a, A, C, and p (see [25])

  *if*_*1*_ *(*p *< 0*.*5)*

   *if*_*2*_ *(|A|* < 1*)*

    Update the position of the current search agent *D* = |*CX*_(*t*)_ −*X*_(*t*)_|

    as a function of inertia weight ‘w’

   *else if*_*2*_ *(|A|* ≥ 1*)*

    Modernize the position of the current search agent by

    *X*(*t* + 1) = *X*_*random*_ − *DA*

   *end if*_*2*_

  *else if*_*1*_ *(p* ≥ 0.5*)*

    Modernize the position by *X*(*t* + 1) = *D e*^*bl*^ *cos*(2*πl*) + X* (*t*) as a function of weight (w)

   *end if*_*1*_

  *end for*

 Check if any search agent goes beyond the search space and amend it.

 Calculate the fitness of each search agent.

 Update the best search agent if there is a better solution.

 *t* = *t* + 1

*end while*

return

where *t* is the No. of iterations, *A* and *C* are the coefficient vectors, X* is the best position (updated every iteration if there is another best solution), *X* is the position vector, and *| |* is the absolute value. *B* is a constant to define the shape of the logarithmic spiral, and *l* is a random number within the interval [*−*1, 1]

### 3.2. For BE modulation

**Fig 3:**
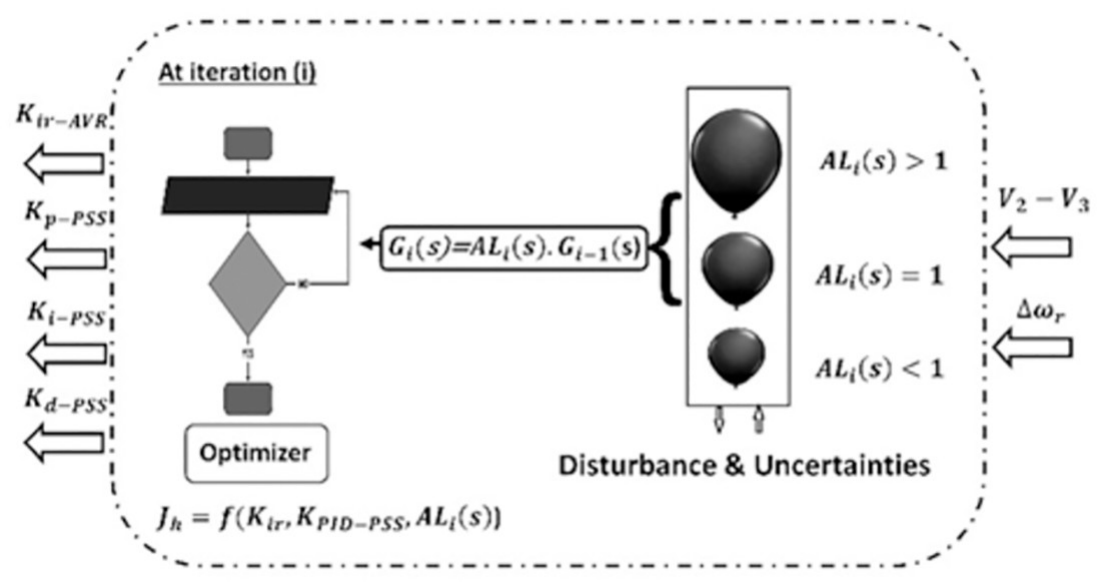
The idea of BE modulation at iteration (i).

For standard optimizers, the desired functionality depends only on the controller’s gain value [31,32]. This means that any changes in the system do not affect the objective function, resulting in poor performance against system uncertainties. Therefore, BE modulation is the solution because it bears the meaning of system variations efficacy on the desired value and thus on the performance of the optimizer itself. The BE concept is described in Fig 3. It is seen that the input *U*_*i*_(*s*) and the output *Y*_*i*_(*s*) are fed into the optimizer to determine the actual transfer function at iteration (i) as:

$$G_{i}\left(s\right)=Y_{i}\left(s\right)/U_{i}\left(s\right)$$ (6)

Moreover, *G*_*i*_(*s*)can be defined based on the previous value *G*_*i*−1_(s) using the parameter coefficient (*AL*_*i*_), as shown in (7):

$$G_{i}\left(s\right)={(AL}_{i}){(G}_{i-1}\left(s\right))$$ (7)

Where, *G*_*i*−1_(s) represents the previous value of the nominal transfer function *G*_o_(s), which can be described as:

$$G_{i-1}\left(s\right)=\left(\rho_{i}\right)(G_{o}\left(s\right))$$ (8)

where $\rho_{i}=\prod_{n=1}^{i-1}{AL}_{n}$

where *V*_2_ and *V*_3_ are forward and feedback AVR voltages. *K*_*ir-AVR*_ *is* the AVR integral controller gain and *K*_*i-PSS*_, *K*_*p-PSS*_, *K*_*d-PSS*_ are gains of the controller of PSS. The flowchart in Fig 3 is typically the proposed EWOA pseudo code, which discussed in the previous subsection 3.1.

## 4. OTEC system design using direct adaptive concept

### 4.1. SMC based rotor speed control system

SMC is a consequence of discontinuous control and compensates for system variations. It operates on an error signal (*X*) and employs a sign function (*sgn*(∙)) and linear filter (*f*_1_) to produce a control input (*U*) [33]. The control input can be represented as:

$$U=\left|f_{1}(X,\dot{X},\ldots ..\right|sgn\left(X,\dot{X},\ldots ..\right)$$ (9)

where |∙| denotes the absolute value, *f*_1_ is an appropriate linear filter and *sgn*(∙) is a sign function defined as:

$$Usgn\left(f\right)=\left\{\begin{array}{ll} 1 & if\;f>0\\ 0 & if\;f=0\\ -1 & if\;f<0 \end{array}\right.$$ (10)

For instance, a modified form of the classical PD corrector is given by:

$$UU=-\left|X\right|sgn\left(X+k\dot{X}\right)$$ (11)

In this study, SMC is used with an estimator for load disturbance to enhance the SC-OTEC performance. Supposing a linear combination function of the rotor speed error and its integration represents the sliding surface as:

$$S(e)=e_{\omega }+\lambda \int e_{\omega \;}dt$$ (12)

$$e_{w}=\omega_{r}-\omega_{r}^{*}$$ (13)

where *e*_*w*_, *w*_*r*_, $w_{r,}^{*}$ represent the tracking error, actual and reference values of the SG rotor speed, and *λ* is constant (> 0) representing the sliding surface slope. By making the time derivative of *S* in (12), it can be found that:

$$\dot{S}=\dot{e_{\omega }}+\lambda e_{\omega }=\dot{\omega_{r}}-\dot{{\omega_{r}}^{*}}+\lambda e_{\omega }$$ (14)

By combining (11) and (14):

$$\alpha \dot{S}=-\alpha (\dot{{\omega_{r}}^{*}}-\lambda e_{\omega })-\beta \omega_{r}-\delta P_{L}+u_{P}$$ (15)

where *α* = 1/*K*_*PID-PSS*_, *β* = *T*_*w*_/*K*_*PID-PSS*_, and *δ* = 1.

To deal with chattering phenomena, an adaptive law is incorporated with SMC to estimate the load disturbance and eliminate undesired chattering effects. Assuming that (*P*_*L*_) is a function of (*ω*_*r*_), as follows.

$$P_{L}=K_{L}.p_{d}\left(\omega_{r}\right)$$ (16)

where *K*_*L*_, *p*_*d*_(*ω*_*r*_) mean unknown constant, known bounded function. Thus, Eq (14) can be rewritten using (15) as:

$$\dot{\alpha S}=-\alpha (\dot{{\omega_{r}}^{*}}-\lambda e_{\omega })-\beta \omega_{r}-\gamma f_{d}\left(\omega_{r}\right)+u_{P}$$ (17)

where *γ* = *K*_*L*_ ⋅ *δ*. Considering nominal parameters case, so *α* = *α*_*n*_, and *β* = *β*_*n*_

At the moment of uncertainties occurring, i.e. the system parameters deviate from their nominal values, so:

$$\alpha =\alpha_{n}+\Delta \alpha \;and\;\beta =\beta_{n}+\Delta \beta$$

In this study, the control effort (*u*_*P*_) is expressed as (18):

$$u_{P}=u_{P-eq}+\Delta u_{P}$$ (18)

Where *u*_*P-eq*_ represents an equivalent control and calculated from the solution of $\dot{S}=0$ under *α* = *α*_*n*_ and *β* = *β*_*n*_. That is:

$$u_{P-eq}=\alpha_{n}(\dot{{\omega_{r}}^{*}}-\lambda e_{\omega })+\beta_{n}\omega_{r}+\hat{\gamma }p_{d}\left(\omega_{r}\right)$$ (19)

where $\hat{\gamma }$ is the predestined parameter to remove the external disturbance. The sliding surface is added to the equivalent control to enhance robustness and durability, as shown in (20).


$$u_{P-eq}=\alpha_{n}(\dot{{\omega_{r}}^{*}}-\lambda e_{\omega })+\beta_{n}\omega_{r}+\hat{\gamma }f_{d}\left(\omega_{r}\right)-kS$$ (20)

The influence caused by system parameter variations is addressed byΔ*u*_*P*_ in (21).


$$\begin{array}{ll} \Delta u_{P}= & -[\text{sup}\left|\Delta \alpha \right|]\;\left(\dot{\omega_{r}{}^{*}}-\lambda e_{\omega }\right).sgn\;\left(S\left(\dot{\omega_{r}{}^{*}}-\lambda e_{\omega }\right)\right)\\ & -[\text{sup}\left|\Delta \beta \right|]\omega_{r}.sgn\;\left(S\omega_{r}\right) \end{array}$$ (21)

In this study, the unknown external disturbance is estimated using an adaptive law in (22), and the stability of the proposed SMC is checked using the Lyapunov function in (23), as follows.

$$\dot{\hat{\gamma }}=-k_{1}\left(\dot{Sp_{d}}\right)-k_{2}\left(Sp_{d}\right)$$ (22)

$$Ϭ\left(t\right)=\frac{1}{2}\alpha S^{2}+\frac{1}{2k_{2}}{\left(\tilde{\gamma }+k_{1}\;Sp_{d}\right)}^{2}$$ (23)

Where $\tilde{\gamma }=\hat{\gamma }-\gamma \;$ and *k*_1_, *k*_2_ are positive constants. The time derivative of the Lyapunov function $\dot{Ϭ}\left(t\right)$ is computed in (24).


$$\dot{Ϭ}\left(t\right)=\alpha S\dot{S}+\frac{1}{2k_{2}}\left(\tilde{\gamma }+k_{1}Sp_{d}\right).\left[\dot{\tilde{\gamma }}+k_{1}\left(\dot{Sp_{d}}\right)\right]$$ (24)

By substituting (18), (21), and (22) into (23) we have:

$$\dot{Ϭ}\leq -kS^{2}-k_{1}S\;f_{d}^{2}\leq -kS^{2}\leq 0$$ (25)

It is shown that $\dot{Ϭ}\left(t\right)\leq 0,$ indicating asymptotic stability of the sliding mode control system.

### 4.2. For PID-PSS controller

**Fig 4:**
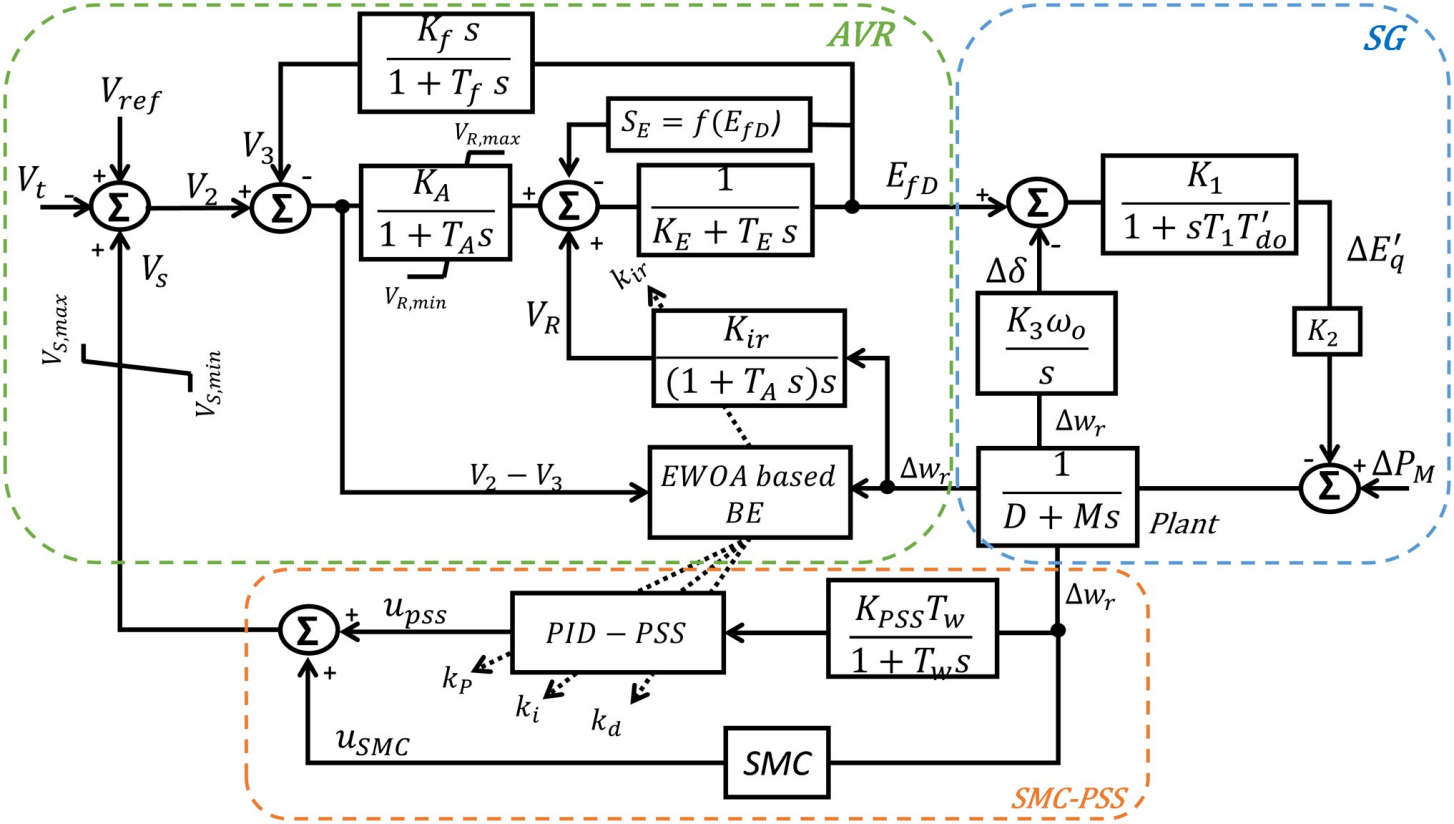
Overall control system diagram-based SMC and BE modulation.

The PSS plays a crucial role in enhancing the grid stability and performance [17]. It serves as an ancillary equipment associated with the excitation system to evolve additional stability constraints on the system. In this study, a PID controller is used for its robust performance and ease of implementation, and it is adaptively tuned using the EWOA optimizer-based BE. The PSS feds by (Δ*ω*_*r*_), which comes directly from the SG. Fig 4 illustrates the overall excitation system block diagram, which includes the secondary AVR and PID-PSS, along with the inclusion of BE and SMC.

A detailed representation of the linearized synchronous machine model can be found in [34], and the state-space equations can be expressed as:

$$\left.\begin{array}{ll} \frac{dx\left(t\right)}{dt} & =Ax\left(t\right)+Bu\left(t\right),y\left(t\right)=Cx\left(t\right)\\ x\left(t\right) & ={\left[\Delta \delta \left(t\right)\Delta \omega \left(t\right)\Delta E_{q}^{\text{'}}\left(t\right)\Delta E_{fd}\left(t\right)\right]}^{T} \end{array}\right\}$$ (26)

where *x*(*t*), *u*(*t*) are the state vector and control input, *y*(*t*) is the control output, and A, B, and C are the matrices of appropriate dismissions. Furthermore, the mathematical model of the proposed PID-PSS is given as follows:

$$V_{s}=\left(\frac{T_{w}s}{\left(1+T_{w}s\right)}\left(K_{P}+\frac{K_{i}}{s}+K_{D}.s\right)\right)\times K_{PSS}\Delta \omega_{r}\left(s\right)$$ (27)

where *K*_*PSS*_ symbolize stabilizer gain and *K*_*P*_, *K*_*i*_ and *K*_*D*_ are the proportional, integral, and derivative controller gains, respectively. These gains are adaptively tuned using the proposed strategy. *T*_*w*_ denotes the washout time constants in sec.

The main target in this study is to mimic the SC-OTEC system response indices, including maximum overshoot (*M*_*P*_), settling time (*T*_*s*_), rise time (*T*_*r*_), and steady-state error (*e*_*ss*_). These indices have been selected as the target performance metrics., as follows.


$$J_{h}=min\sum \left(T_{r}+T_{s}+M_{P}+e_{ss}\right)$$ (28)

In order to calculate these indices, we should first represent the actual transfer function of the 4^th^-order excitation model using the Heffron-Philips’s mode [35] as follows:

For an open loop:

$$G_{plant}=\frac{\Delta \omega_{r}\left(s\right)}{{\Delta V}_{ref}\left(s\right)}=\frac{-ms}{h_{4}s^{4}+h_{3}s^{3}+hs^{2}+hs+h_{0}}$$ (29)

Where,

$$\left.\begin{array}{c} m=k_{E}k_{2}k_{3}\\ h_{4}=Mk_{3}T_{do}^{\text{'}}T_{E};h_{3}=M\left(k_{3}T_{do+}^{\text{'}}T_{E}\right)\\ h_{2}=M+314K_{1}k_{3}T_{do}^{\text{'}}T_{E}+k_{E}k_{6}k_{3}M\\ h_{1}=314K_{1}(k_{3}T_{do+}^{\text{'}}T_{E})-314k_{2}k_{3}k_{4}T_{E}\\ h_{0}=314(K_{1}-k_{2}k_{3}k_{4}-k_{E}k_{2}k_{3}k_{5}-k_{E}k_{1}k_{3}k_{6}) \end{array}\right\}$$ (30)

These coefficients mainly depend on the nominal inertia of the synchronous machine and time constants. Thus, the transfer function in this case can be expressed as in (31). In most cases, instability problems happen due to open-loop systems. Hence, it is necessary to design and incorporate a robust PID-PSS to mitigate these problems and transfer the system into a closed-loop one. Consequently, the final actual transfer function for the proposed system, denoted as *G*_*CL*_(*s*,*m*,*h*), can be described by (32).

$$G_{OL}\left(s,m,h\right)=\frac{-\left[m_{11},m_{12}\right]s}{\left[h_{41},h_{42}\right]s^{4}+\left[h_{31},n_{32}\right]s^{3}+\left[h,n_{22}\right]s^{2}+\left[h_{11},h_{12}\right]s+\left[h_{01},h_{02}\right]}$$ (31)

GCLs,m,h=-m11,m12sh41,h42s4+h31,h32s3+h21+m11kd,h22+m21kds2+h11+m11kp,h12+m21kps+h01+m11ki,h02+m12ki (32)

Where,

$$\begin{array}{c} h_{i1}=\overset{min}{\overbrace{p,Q}}\left(h_{i}\right);h_{i2}=\overset{max}{\overbrace{p,Q}}\left(h_{i}\right);m_{11}=\overset{min}{\overbrace{p,Q}}\left(m\right);m_{12}=\overset{max}{\overbrace{p,Q}}\left(m\right)\\ i=0,1,2,3,4 \end{array}$$

To promote a control constrained-based direct adaptive PID-PSS for a large operating grid, necessary and sufficient constraints need to be applied as follows [35]:

h41>0,h31>0,h21+m11kd>0h11+m11kp>0,h01+m11ki>0h01*h32+m11h32kih21*h11+m11kd*h21*m11kp+m211kpkd<0.465h21*h31+m11h31kdh12*h42+m12h42kp<0.4655

To calculate the objective *J*_*h*_, we must determine the natural frequency (*ω*_*n*_) and damping ratio (*ζ*). It is noted from (32) that the system order is four, so we need to factorize the denominator to a second-order form. This can be achieved using the following simple pseudo-code in MATLAB:

$$sys=tf\left(-\left[m_{11},m_{12}\right],[\left[h_{41},h_{42}\right]\left[h_{31},h_{32}\right]\left[h_{21}+m_{11}k_{d},h_{22}+m_{21}k_{d}\right]\left[h_{11}+m_{11}k_{p},h_{12}+m_{21}k_{p}\right]\left[h_{01}+m_{11}k_{i},h_{02}+m_{12}k_{i}\right]\right)$$

then finding the poles and zeros as:

poles=roots(cell2mat(sys,Den)

zeros=roots(cell2mat(sys,Num)

So, it will be easy to determine the indices of parameters using the standard second-order formula as follows:

GCLs=ωn2s2+2ζωns+ωn2Ts=4ωnζandTr=π-1-ζ2ωn1-ζ2MP=e-πζ1-ζ2,ess=lims→0sΔVref(s)GCL(s,m,n).fVs (33)

It is observed from (33) that parameter indices function in the stabilizer voltage (*V*_*s*_), indicating that *ω*_*n*_ and *ζ* are functions of the PID-PSS gain (*K*_*PID-PSS*_). In this work, the PID-PSS gain is assumed to be adaptively adjusted based on the BE modulation.

### 4.3. Secondary AVR based EWOA

#### A. Without BE modulation

**Fig 5:**
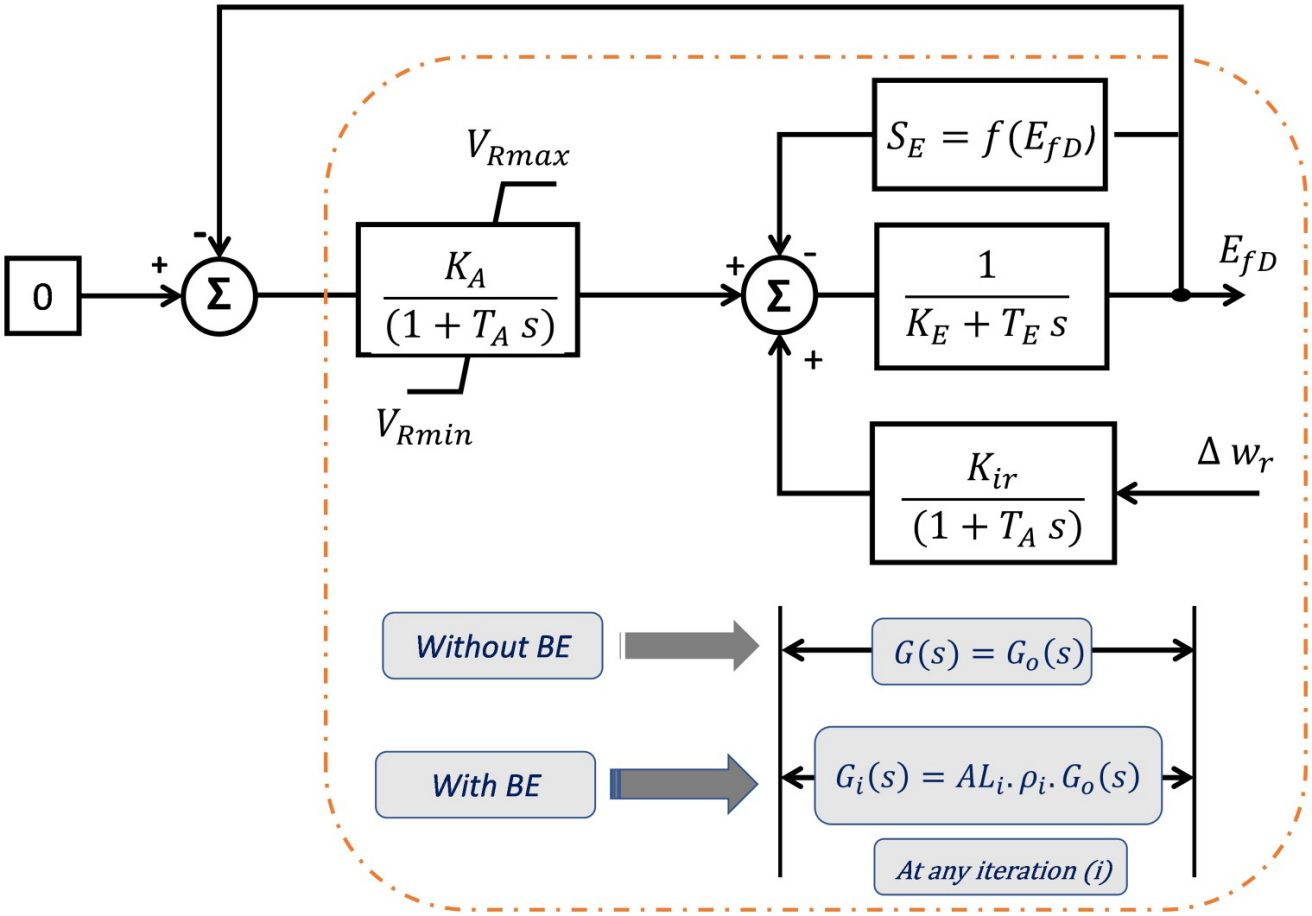
Reduced system model with/without balloon effect.

In this part, a superposition theory is proposed to calculate the closed-loop plant considering Δ*ω*_*r*_ as the excitation system input signal, as shown in Fig 5. The classic EWOA is used for the adaptive secondary AVR design, which tunes the integral controller gain (*K*_*ir*_) based on the speed deviation signal. For simplification, (*S*_*E*_) is neglected.

Gos=KEo+KAoTAoTEos2+TAoKEo+TEoTAoTEos+KEo+KAoTAoTEoωn=KEo+KAoTAoTEo,ζ=TAoKEo+TEo2TATEKEo+KAoTAoTEoTso=8TAoTEoTAoKEo+TEoandTro=π-1-ζ2ωn1-ζ2MPo=e-π*TAoKEo+TEo2TATEKEo+KAoTAoTEo1-TAKE+TE2TAoTEoKEo+KAoTAoTEo2 (34)

Where 0 denotes a nominal value of the parameter.

At iteration (i), the objective function of the classic EWOA depends only ${G_{CL}}_{0}(s)$ (nominal system parameters). This means that it does not account for parameter variations, which is a weakness of the classical EWOA. Also, *K*_*ir*_ = *η*(*K*_*E*0_ + *K*_*A*0_) where *η* is a constant value.

#### B. With BE modulation

Fig 5 shows a simplified excitation system model with the suggested EWOA-based BE modulation. It is used to determine the parameters of the 2^nd^ order closed-loop system. At any iteration (i), the following can be observed:

$${G_{CL}}_{i}\left(s\right)={(AL}_{i})\rho_{i}{G_{CL}}_{o}\left(s\right)$$ (35)

Where, ${G_{CL}}_{o}\left(s\right)means\;the\;system\;actual\;plant$, and

$${\omega_{n}}^{2}=\frac{K_{ir}{AL}_{i}\;\rho_{i}}{T_{A0}T_{E0}}\;and\;2\zeta \omega_{n}=\frac{T_{A0}K_{E0}+T_{E0}}{T_{A0}T_{E0}}$$ (36)

So,

$$J=f\left(K_{ir},{AL}_{i,}{\;\rho }_{i}\right)$$ (37)

It is now clear that (J) is a function in *K*_*ir*_ and *AL*_*i*_ at iteration (i). This means that any change in the system parameters will instantly affect the coefficient value (*AL*_*i*_), resulting in a variation in the objective function at this step. This enhances the EWOA optimizer’s ability to suppress difficulties in the studied SC-OTEC system. To maintain system stability, *K*_*ir*_/*K*_*PID*_ should be ≤ 2*ηω*_*n*_. The robustness and stability validation of the proposed adaptive control with the help of BE modulation is further clarified in [31].

In this work, it is assumed that any deviations in the power system parameters are immediately detectable by the controller in a very short time. The reason behind this that in realistic applications, two Phasor Measurement Units (PMUs) have been deployed [36]. PMUs play a key role in minimizing detection time by providing high-speed synchronized measurements of voltage and current phasors. In our studied SC-OTEC system, we strategically deployed two PMU units—one positioned at the grid side and the other at the SC-OTEC side. The synchronized measurements from PMUs facilitate accurate and time-stamped data, allowing the adaptive control strategy to respond swiftly to changes in the system’s operating conditions.

## 5. Dynamic response simulations

In this section, a dynamic simulation of the proposed grid-connected SC-OTEC system is performed. The simulation aimed to validate the proposed strategy’s efficacy in dampening oscillations caused by solar radiation (*S*) and surface seawater temperature (*T*_*a*_) variations. The SC-OTEC system is tested under the following study cases:

### 5.1. Special case study: a) long-term variations in solar radiation

**Fig 6:**
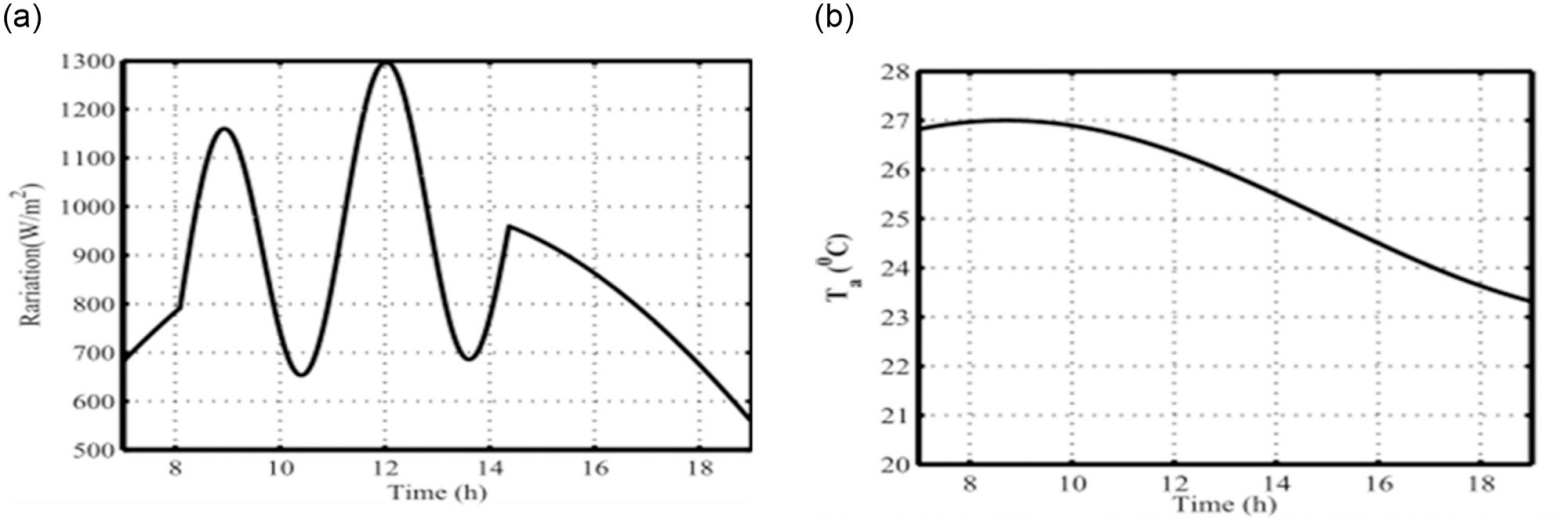
(a) Solar radiation; (b) Ambient temperature data.

This scenario highlights the dependence of solar radiation change on the real power of the OTEC system. Randomly measured solar radiation data (W/m^2^) are used to imitate the system’s changes (with bound of |ΔRadiation| = 30% rated value). Long-term variations in solar radiation are applied to the SC-OTEC system. Fig 6 shows the recorded variations and temperatures from 9:00 to 15:00 in hours.

**Fig 7:**
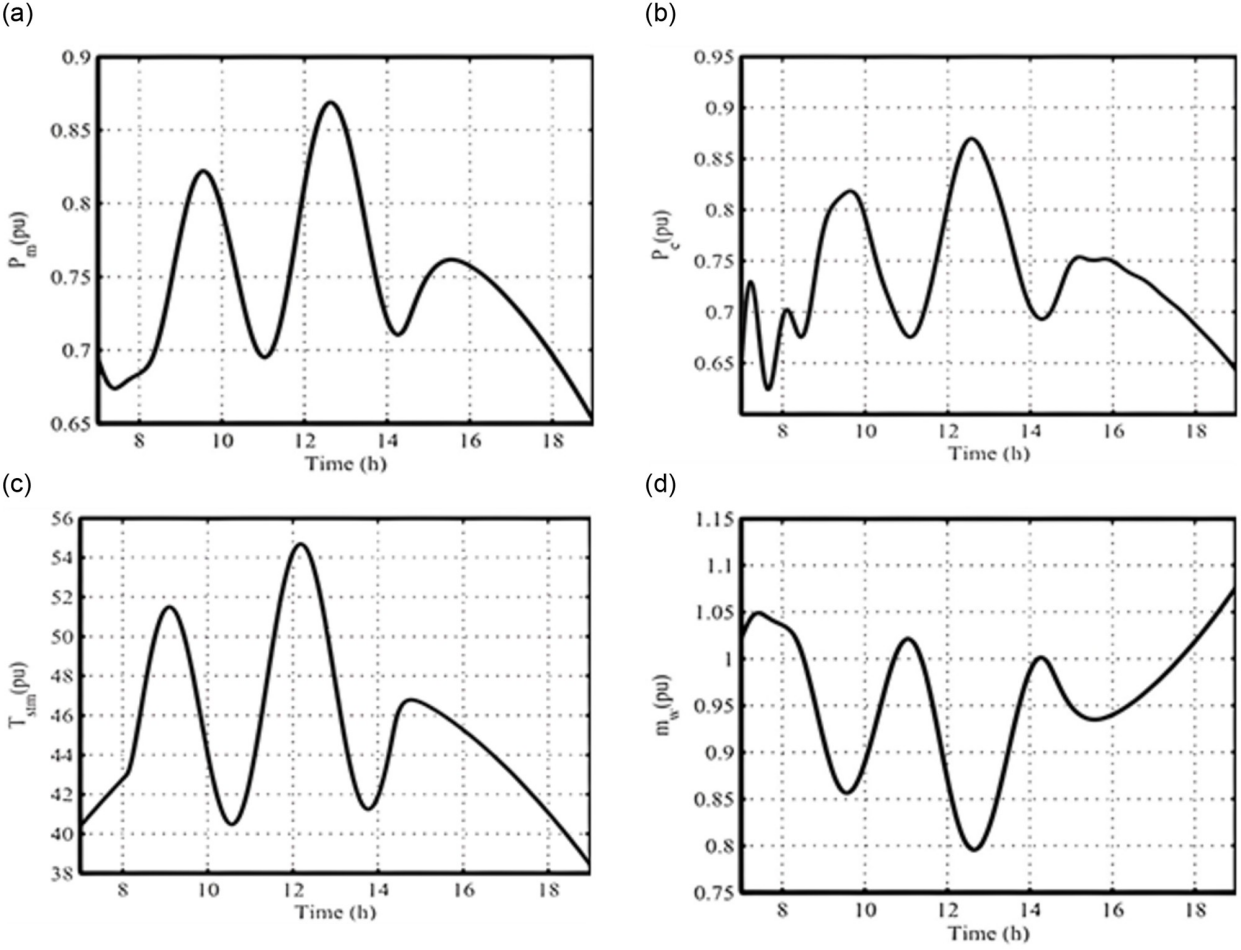
Dynamic transient responses of the SC-OTEC system under a change in solar radiation (a) P_m_; (b) P_e_; (c) T_stm_; (d) m_w_.

With the centralized solar system, the temperature difference between the condenser and the evaporator can be increased, raising the ammonia vapor pressure to wheels up the turbine blades. The resulting 7-h responses for the studied SC-OTEC system are presented in Fig 7(a)–7(d), using a simulation time step of one second. Due to the gradual changes in the employed solar radiation, the deviations in the rotor speed Δ*ωr*, and the terminal voltage Δ*V*_t_ of the SG remain zero due to the impact of the excitation and governor systems operation.

The generated turbine power (*P*_*m*_) in Fig 7a, the grid’s active power (*P*_*e*_) in Fig 7b, the output heat exchanger temperature 〖*T*_*stm*_) in Fig 7c exhibit similar patterns to the solar radiation change shown in Fig 6. However, the mass flow rate of the warm seawater *m*_*w*_ in Fig 7d shows a different response. The addition of classic EWOA with PSS and AVR, along with SMC, is only active during system perturbation conditions. Therefore, it does not contribute to the effective damping properties of the SC-OTEC during slow variations in solar radiation.

### 5.2. The effect of changes in surface seawater temperature

**Fig 8:**
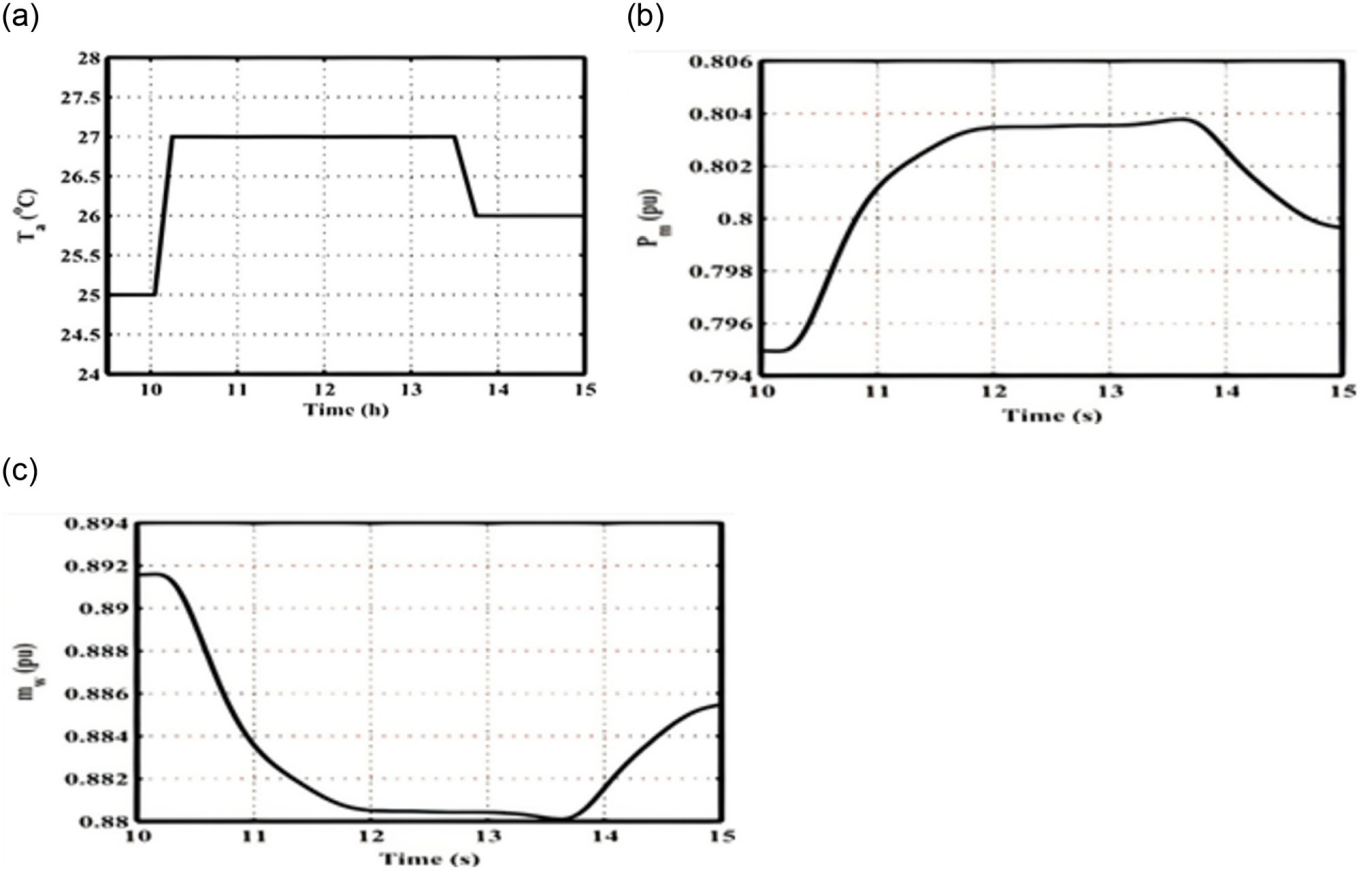
OTEC system dynamics responses considering a change in surface water temperature (a) Step change variations in ambient temperature; (b) *P*_*m*_; (c) *m*_*W*_.

This scenario demonstrates how changes in the surface sea temperature affect the real OTEC output power. As solar insolation increases gradually, the temperature of the surface seawater rises as well. According to the heat exchanger equation, a 1°C increase in surface seawater temperature results in approximately a 1°C increase in the average heat exchanger temperature. Consequently, the real power output rises as well. Therefore, this scenario illustrates the impact of changing the surface seawater temperature *T*_*a*_ (with bound of |ΔT_a_ | = 10% rated value) on the real OTEC output power. For further clarification, Fig 8a shows a step change in the ambient temperature (*T*_*a*_) applied to the OTEC Simulink model while keeping the solar insolation at 1000 W/m^2^. Fig 8(b) and 8(c) depict the observed variations during the 10–15 hours period. The findings indicate a direct correlation between the output real power (*P*_*M*_) and mass flow rate (*m*_*w*_) with changes in the surface temperature.

### 5.3. Analysis of SC-OTEC system with/without balloon effect modulation and SMC

The studied grid-connected SC-OTEC system was investigated to assess the impact of load changes, surface sea temperature variations, and 3*ϕ*-SC sever fault at the grid bus. The objective was to validate the superiority of the proposed control strategy (SMC combined with adaptive PID-PSS and secondary AVR based on EWOA-based BE) in suppressing fluctuations compared to the conventional SC-OTEC system (no PSS, no SMC, no EWOA, no BE) and a system with PSS only (Kunder). Two scenarios were tested to evaluate the effectiveness of the proposed control strategy, as follows.

#### A. Scenario 1: The effect of a 3*ϕ* short-circuit fault at the grid AC bus

In this scenario, the proposed control strategy for SC-OTEC was investigated after a 3*ϕ*-SC sever fault occurred at the grid AC bus, as shown in Fig 1. The fault starts at t = 10 s and lasts for 0.07 s, within a total simulation time of 15 seconds. The objective of this study is to compare the performance of the proposed direct adaptive PID-PSS and secondary AVR with SMC and BE modulation via the conventional SC-OTEC system, as well as the system with PSS within the nonlinear exciter system without BE.

**Fig 9:**
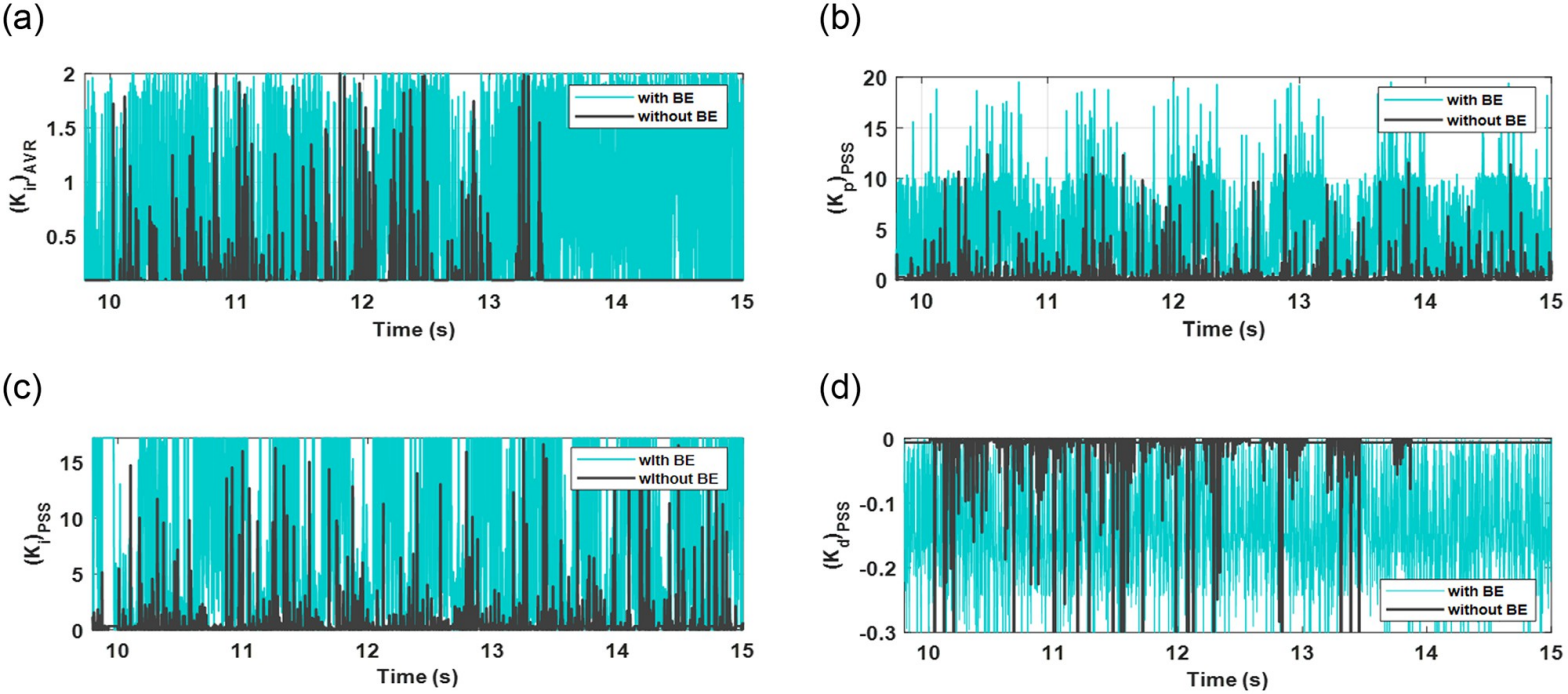
Optimizer output control signals for integral gain for secondary AVR integral gain and PID controller gains for PSS: (a) K_ir_; (b) K_i_; (c) K_p_; (d) K_d_.

Fig 9 shows the output optimizer control signal of the secondary AVR integral controller gain (*k*_*ir*_) and PID-PSS control gains (*k*_*PID-PSS*_) using 50 iterations and 5 candidate solutions to optimize the function (*J*_*h*_) described in (28). It can be observed that the addition of BE helps the EWOA optimizer to perform more efforts during contingencies, making the algorithm more sensitive and traceable against uncertainties.

**Fig 10:**
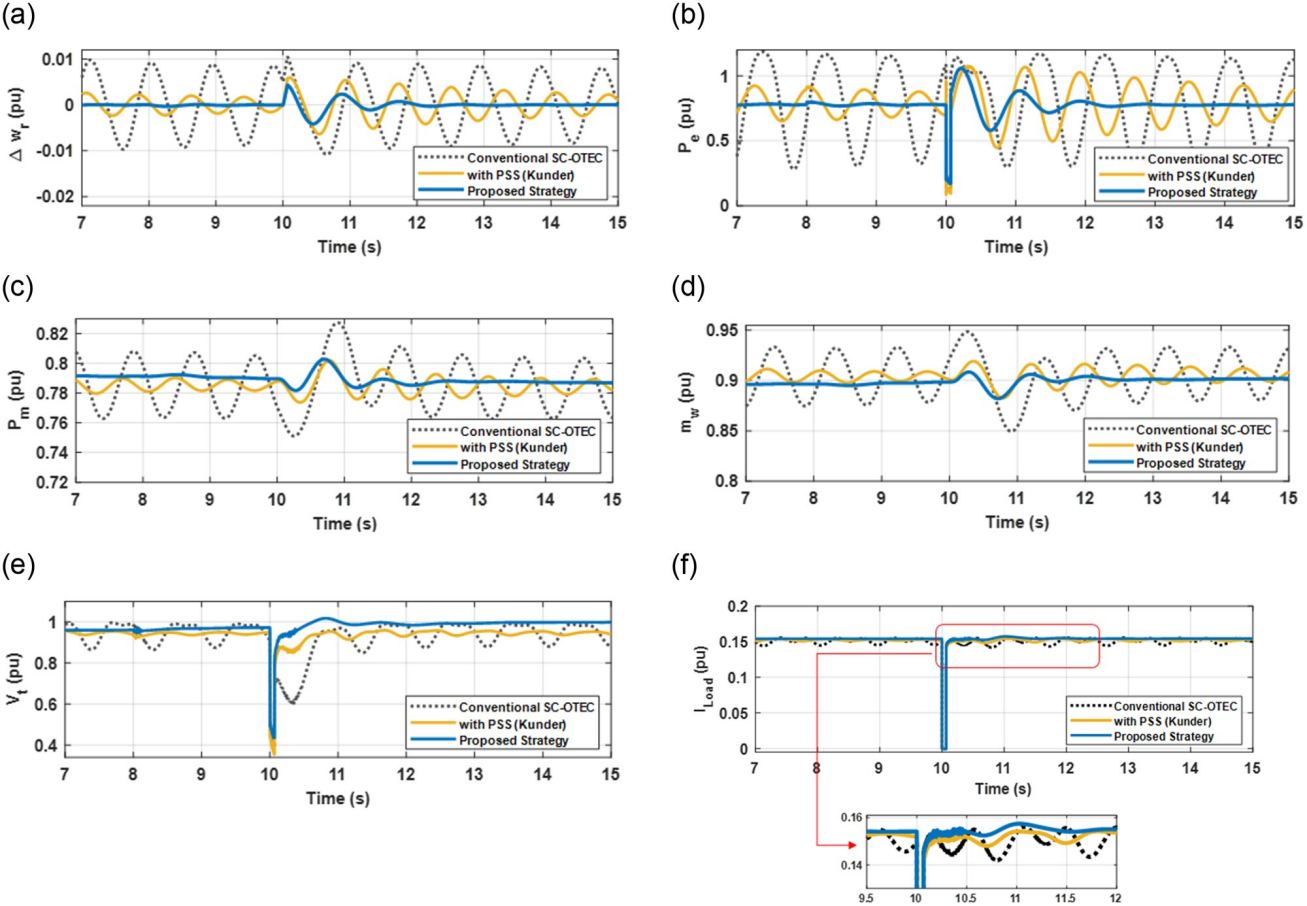
Transient responses of the SC-OTEC tied to grid considering a 3*ϕ*-SC fault (a) Δw_r_; (b) P_e_; (c) P_m_; (d) m_w_; (e) V_t_; (f) I_Load_.

Fig 10(a)–10(e) demonstrate that the vibrations of the SC-OTEC system are effectively damped and suppressed when the excitation system is equipped with a secondary AVR and PID-PSS using the EWOA optimizer-based BE with SMC. It is noted from Fig 10 that much improvements in the dynamic response of the electrical power (*P*_*e*_), mechanical power (*P*_*M*_), mass flow rate component (*m*_*w*_), terminal voltage (*V*_*t*_), load current(*I*_*load*_), and SC-OTEC frequency are evident in terms of (*T*_*s*_, *M*_*P*_, *e*_*ss*_), with the inclusion of the proposed strategy. These findings highlight a supremacy performance in damping oscillations achieved by the adaptive direct application of BE-modulated PID-PSS with SMC and secondary AVR in the SC-OTEC system.

#### B. Scenario 2: The effect of switching local residential loads

**Fig 11:**
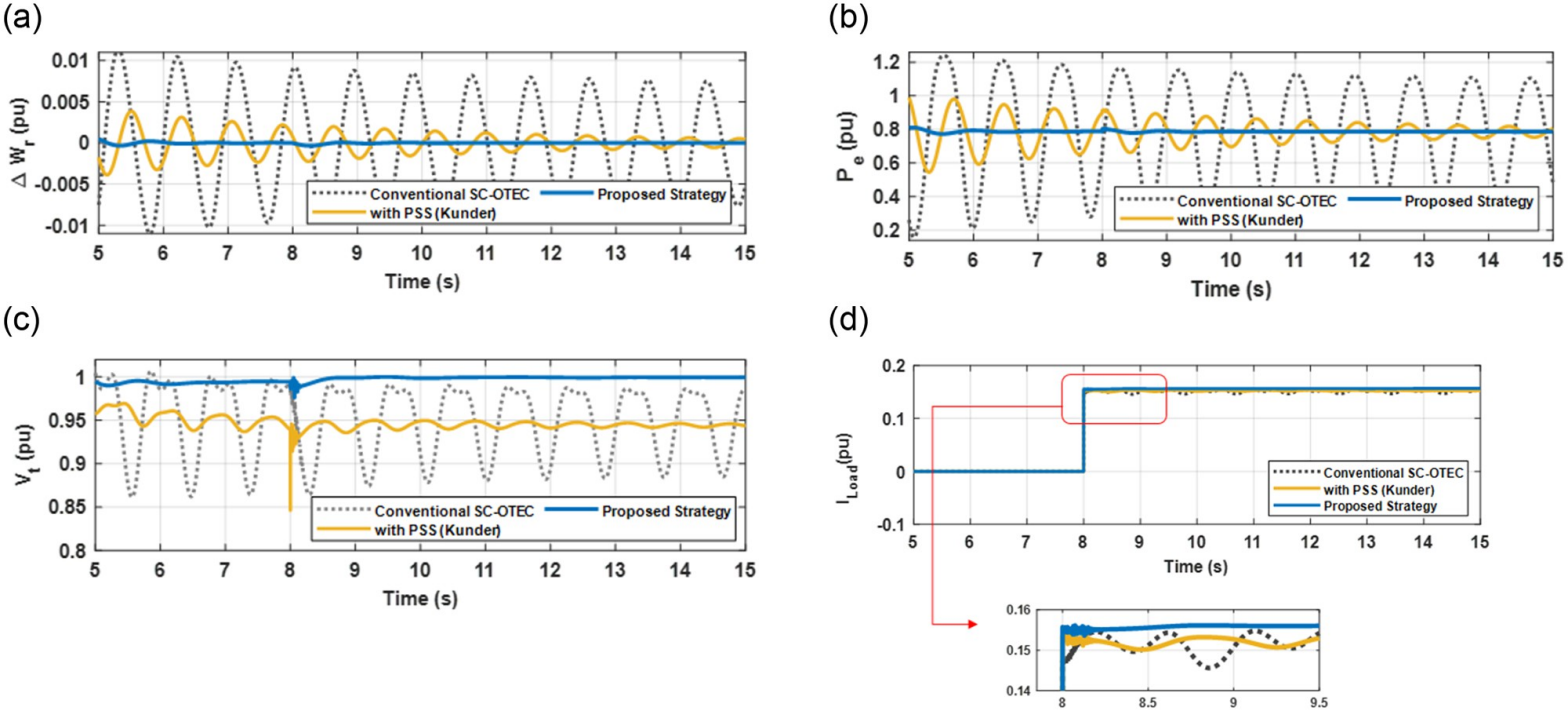
The considered grid-connected SC-OTEC system responses considering a change in loads near the power grid (a) Δ*w*_*r*_; (b) *P*_*e*_; (c) *V*_*t*_; (d) *I*_*Load*_.

In this scenario, three local loads connected to the grid were chosen for examination as shown in Fig 1. The total load consists of two units with a combined power of 20 MW (resistive and inductive) and one unit with a power of 0.5 MW (capacitive). Fig 11 describes the studied grid-connected SC-OTEC system responses considering a change in loads, which starts from t = 8 s and continues for 7 s, followed by the load disconnection. A comparative study on the performance of the proposed strategy under the influence of connected/disconnected loads using circuit breakers (CBs) was investigated in this study.

It is observed from Fig 11 that the operating point for the SC-OTEC system with Kunder (PSS) starts from 0.95 pu and not reach the pre-defined value (1 pu). This is due to the controller’s response time and adaptive capabilities. Both the conventional SC-OTEC system and the system with PSS only experience undesirable deviations leading to system instability. In contrast, when an adaptive PID is added to the PSS and associated with a secondary AVR within a nonlinear exciter system with the help of SMC, superior robustness is achieved, effectively addressing this contingency. The electro-oscillations caused by the switching mechanism are damped within approximately 1 second when the excitation system is equipped with the suggested control strategy. Hence, the direct adaptive concept utilizing the EWOA optimizer-based BE modification, when combined with PID-PSS and SMC, along with a secondary AVR, effectively dampens oscillations and ensures stability in the SC-OTEC system tied to the grid.

## 6. Conclusion

This This paper introduces a new control strategy for an offshore SC-OTEC system connected to an onshore grid. The proposed strategy combines SMC with adaptive PID-PSS and secondary AVR to enhance system performance and damping. The key idea is to utilize the BE modulation with EWOA to self-tune the controller gains for the auxiliary secondary AVR and PID-PSS.

The performance of the studied SC-OTEC system is extensively examined through time domain simulations, considering long-term variations in solar irradiance and surface seawater temperature. Comparative analysis is also conducted under severe 3*ϕ*-SC fault and load changes scenarios. The results demonstrate that the proposed control strategy maintains a terminal voltage operating point of 1 pu during load changes, outperforming the conventional approach (0.95 pu). Additionally, the proposed strategy exhibits superior damping with a rotor speed deviation of ±0.0005 pu during fault compared to ±0.001 pu in the conventional one. These findings highlight the significant enhancement the OTEC plant’s thermal efficiency, especially during daytime operation, while ensuring the dynamic stability of the main AC grid.

In future research, the proposed control strategy will undergo experimental validation to examine how surface seawater temperature and solar insolation affect the net output power of the OTEC system. Additionally, a comparative analysis will be conducted to assess the superiority of the proposed strategy against other robust controllers, including coefficient diagram and H-infinity methods.

## Supporting information

S1 Appendix (PDF)

S1 Table Nomenclature. (PDF)

S1 File (M)

S2 File (XML)

S3 File (RELS)

S4 File (XML)

S5 File (XML)

S6 File (PNG)

S7 File (RELS)

S8 File (MAT)

S9 File (XML)

S10 File (ZIP)
