# Supplementary material for: Robust SMC-PSS and AVR design: A grid connected solar concentrated OTEC system application
Source: PLoS One. 2023 Dec 22;18(12):e0295941. doi: 10.1371/journal.pone.0295941 (PMC10745166; doi:10.1371/journal.pone.0295941)
Supplement: S1 Appendix — (PDF) [file pone.0295941.s001.pdf]

**Table 1.** Initial parameters of the studied single machine infinite bus system.

| Synchronous generator | | Value |
|---|---|---|
| Nominal power (MVA), line-to-line voltage (KVrms), and frequency (Hz) | | [100, 13.8, 60] |
| Stator [$R_s$, $L_1$, $L_{md}$, $L_{mq}$] (pu) | | [0.00285, 0.114, 1.19, 0.36] |
| Field [$R_f$, $L_{1fd}$] (pu) | | [0.000579, 0.114] |
| Inertia coefficient, friction factor, poles [H(s)F(pu), p()] | | [3.7, 0, 20] |
| **Three-phase Transformer** | | |
| Nominal power and frequency [ $P_n$(MVA), $f_n$(Hz)] | | [100, 60] |
| Winding 1 [ $V_{1Ph-Ph}$(rms), $R_1$(pu), $L_1$(pu)] | | [13.8e3, 0.002, 0.8] |
| Winding 2 [ $V_{2Ph-Ph}$(rms), $R_2$(pu), $L_2$(pu)] | | [230e3, 0.002, 0.8] |
| **Excitation system** | | |
| Time constant of low-pass filter $T_r$(s) | | 0.02 |
| Regulator gain and time constant [$K_a$(), $T_a$(s)] | | [300, 0.001] |
| Exciter gain and time constant [$K_e$(), $T_e$(s)] | | [1, 1e-3] |
| Damping filter gain and time constant [$K_f$(), $T_f$(s)] | | [0.001, 0.1] |
| Regulator output limits and gain [$V_{Rmin}$, $V_{Rmax}$, $K_c$] | | [-2, 2, 1] |
| **Transmission line** | | |
| Line 1 | Resistance (Ohms/km) | 0.0529 |
| | Capacitance (nF/km) | 8.7749 |
| | Line length (km) | 220 |
| Line 2 | Submarine Cable Length (km) | 10 |
| | Cable apparent resistance (Ohms/km) | 0.102 |
| | Cable reactance (Ohms/km) | 0.13 |
| | Cable capacitance (mF/km) | 0.25 |

**Table 2.** Initial SC-OTEC system parameters.

| Parameter | Value | Parameter | Value |
|---|---|---|---|
| $U_L$ | 6 | $\Delta T_{dseign}$ | 2.5 |
| $U_s$ | 3 | $\mu$ | 0.53 |
| $m'_{cs}$ | 5 | $F_d$ | 0.841 |
| $m'_w$ | 2 | $K_{TEM}$ | 1.3 |
| $M_w$ | 12 | $\alpha$ | 0.9 |
| $A_c$ | 2200 | $T_{TEM}$ | 0.01 |
| $A_s$ | 10668.3 | $T_w$ | 25 |
| $M_{w0}$ | 0.885 | $T_c$ | 5 |
| $\tau$ | 0.9 | $T_{ch}$ | 1 |
| $C_w$ | 4190 | $T_1$ | 0.001 |
| $C_p$ | 838 | $T_2$ | 0 |
| $P_{Mo}$ | 0.8 | $T_3$ | 0.01 |
| $K_r$ | 20 | | |
