# Supplementary material for: Robust SMC-PSS and AVR design: A grid connected solar concentrated OTEC system application
Source: PLoS One. 2023 Dec 22;18(12):e0295941. doi: 10.1371/journal.pone.0295941 (PMC10745166; doi:10.1371/journal.pone.0295941)
Supplement: S1 Table — (PDF) [file pone.0295941.s002.pdf]

Nomenclature

OTEC	Ocean thermal energy conversion system	T_c	Depth cold seawater temperature (°C)
EWOA	Enhanced whale optimizer	C_w	Water heat capacity (J/kg)
SMC	Sliding mode control	C_p	Seawater heat capacity (J/(kg.°C))
PSS	Power system stabilizer	S	Solar insolation (W/m²)
AVR	Automatic voltage regulator	α	Absorber pipe dimensionless absorptance
BE	Balloon effect modulation	τ	Glass cover transmittance
q_u	Water heat collected quantity (kW)	F''	Collector efficiency factor
F_R	Dimensional heat removal	μ	Cold to warm water flow rate ratio
A_C	Parabolic mirror aperture area (m²)	ρ	Average density of seawater(kg/m³)
A_S	Heat exchanger surface area in (m²)	ΔP_{net}	Change in net power (W)
U_L	The collector heat loss coefficient	ΔP_M	Change in mechanical power (W)
U_S	Dimensional heat loss coefficient of exchanger collector	K_{TEM}	Controller gain
T_{stm}	Heat exchanger average output temperature	T_{TEM}	Controller time constant (s)
ΔT_{dseign}	Difference of ideal temperature	m_{cs}	Water mass flow rate (kg/s)
T_{wx}	Surface warm seawater temperature (°C)	m_w	Surface warm seawater mass flow rate (kg/s)
T_a	Ambient temperature (°C)	M_w	Heat exchanger fluid mass (kg)
