# Supplementary figures and images for: Robust SMC-PSS and AVR design: A grid connected solar concentrated OTEC system application
Source: PLoS One. 2023 Dec 22;18(12):e0295941. doi: 10.1371/journal.pone.0295941 (PMC10745166; doi:10.1371/journal.pone.0295941)

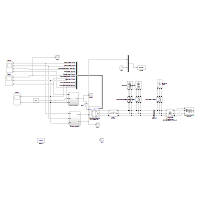

Supplement: S6 File — (PNG) [file pone.0295941.s008.png]
